# Characteristics and outcomes of patients with community-acquired and hospital-acquired sepsis

**DOI:** 10.5935/0103-507X.20190013

**Published:** 2019

**Authors:** Glauco Adrieno Westphal, Aline Braz Pereira, Silvia Maria Fachin, Ana Carolina Caldara Barreto, Ana Carolina Gern Junqueira Bornschein, Milton Caldeira Filho, Álvaro Koenig

**Affiliations:** 1 Centro Hospitalar Unimed Joinville - Joinville (SC), Brasil.; 2 Universidade da Região de Joinville - Joinville (SC), Brasil.

**Keywords:** Sepsis, Iatrogenic disease, Community-acquired infections, Mortality, Brazil

## Abstract

**Objective:**

To compare the clinical characteristics and outcomes of patients with community-acquired and hospital-acquired sepsis.

**Methods:**

This is a retrospective cohort study that included all patients with a diagnosis of sepsis detected between January 2010 and December 2015 at a private hospital in southern Brazil. Outcomes (mortality, intensive care unit and hospital lengths of stay) were measured by analyzing electronic records.

**Results:**

There were 543 hospitalized patients with a diagnosis of sepsis, with a frequency of 90.5 (85 to 105) cases/year. Of these, 319 (58%) cases were classified as hospital-acquired sepsis. This group exhibited more severe disease and had a larger number of organ dysfunctions, with higher hospital [8 (8 - 10) *versus* 23 (20 - 27) days; p < 0.001] and intensive care unit [5 (4 - 7) *versus* 8.5 (7 - 10); p < 0.001] lengths of stay and higher in-hospital mortality (30.7% *versus* 15.6%; p < 0.001) than those with community-acquired sepsis. After adjusting for age, APACHE II scores, and hemodynamic and respiratory dysfunction, hospital-acquired sepsis remained associated with increased mortality (OR 1.96; 95%CI 1.15 - 3.32, p = 0.013).

**Conclusion:**

The present results contribute to the definition of the epidemiological profile of sepsis in the sample studied, in which hospital-acquired sepsis was more severe and was associated with higher mortality.

## INTRODUCTION

Sepsis, which is defined as a life-threatening organ dysfunction caused by a dysregulated host response to infection, is an important public health problem.^(^^1^^)^ Based on population studies conducted in the United States that showed an annual sepsis incidence of 300 cases per 100,000 inhabitants, the estimated incidence in Brazil is 600,000 cases per year, generating massive costs for the healthcare system.^(^^2,3^^)^ In addition to its high incidence, sepsis is a leading cause of death in intensive care units (ICUs) worldwide.^(^^4^^)^ Despite advances in diagnosis and management, sepsis-related mortality continues to be high, especially in developing countries.

In Brazil, the mortality rate is 55.7% according the SPREAD study and 57.4% according to the PROGRESS study,^(^^5,6^^)^ which is in contrast with 45% observed in other developing countries and 38.2% in developed countries.^(^^7,8^^)^

Strategies to reduce sepsis-related mortality are widely disseminated through the Surviving Sepsis Campaign (SSC)^(^^9^^)^ and include fluid resuscitation, lactate measurement and obtaining blood cultures before the administration of antibiotics, as well as the administration of broad-spectrum antibiotics within the first hour of diagnosis. Several studies published over the last 15 years have confirmed that interventions that contribute to a reduction in sepsis-related mortality are early detection, resuscitation and antibiotic therapy.^(^^10-13^^)^ However, these studies mainly evaluated patients seen at emergency departments, which primarily correspond to cases of community-acquired sepsis. A recent study conducted by the *Instituto Latino Americano de Sepse* (ILAS) in Brazilian adult ICUs showed that for patients with hospital-acquired sepsis, the time to diagnosis is longer, adherence to treatment is lower, and mortality is higher.^(^^5^^)^

Few studies, however, have compared the characteristics, evolution and mortality of patients with community-acquired and hospital-acquired sepsis.^(^^14^^)^

Within this context, the objective of this study was to compare the clinical characteristics and outcomes of patients with community-acquired and hospital-acquired sepsis.

## METHODS

This was a retrospective cohort study that analyzed the records of septic patients from a private hospital in southern Brazil identified between January 2010 and December 2015.

All patients older than 18 years diagnosed with sepsis between January 2010 and December 2015 were included. The diagnosis of sepsis was established based on the recognition of a suspicious or confirmed source of infection associated with at least one organ dysfunction. Patients at risk of infection and sepsis were screened based on signs suggestive of infection and clinically detectable organ dysfunction (Table 1).

**Table 1:** Expanded clinical signs of infection, including clinically detectable signs of organ dysfunction

Clinical signs
Axillary temperature greater than 38ºC or less than 36ºC
Heart rate greater than 90 beats per minute
Systolic blood pressure less than 90mmHg or MAP less than 65mmHg
Respiratory rate greater than 20 breaths per minute
Need for oxygen supplementation
Acute encephalopathy (drowsiness, disorientation, confusion, or coma)
Urinary output less than 0.5mL/kg/h

MAP - mean arterial pressure.

The patients were divided into two groups according to the origin of sepsis identified in the medical records: community-acquired sepsis and hospital-acquired sepsis. Cases of sepsis diagnosed on hospital admission or up to 48 hours thereafter were classified as community-acquired and cases diagnosed 48 hours after hospital admission were classified as hospital-acquired. Patients transferred from another hospital, with incomplete records and with end-stage disease according to the judgment of the assisting medical team were not included in the study.

The screening of sepsis consisted of actively looking for signs suggestive of infection and clinically detectable organ dysfunction at the first examination in all patients seen on the wards and in the emergency department. Nurse technicians were trained to identify and communicate the manifestation of two or more signs suggestive of infection to the department nurse. After recording signs suggestive of infection, the electronic medical record was programmed to send electronic alerts to mobile devices carried by the nurses. After initial assessment by the nurse, the medical team was notified to evaluate the patient, to confirm the diagnosis of sepsis, to look for other organ dysfunctions not evaluated in the screening protocol and to initiate treatment.^(^^5,7,9-15^^)^ The diagnosis of sepsis (formerly called *severe sepsis*) was defined in the presence of a presumed infection plus any organ dysfunction. Septic shock was identified by the vasopressor requirement to maintain a mean arterial pressure (MAP) of 65mmHg.^(^^1^^)^

Clinical management was based on the recommendations proposed by the SSC, which included obtaining blood cultures before the administration of antibiotics, antibiotic therapy in the first hour after the diagnosis of sepsis, an initial lactate measurement, adequate volume expansion (defined as the administration of at least 30mL/kg of crystalloid fluid when hypotension or lactate level > 4mmol/L), and the use of vasopressors in the case of persistent hypotension (MAP < 65mmHg). The complete resuscitation bundle was characterized as the accomplishment of all these steps.

The variables studied were sex, age, Acute Physiology and Chronic Health Disease Classification System II (APACHE II) score, type of hospitalization (surgical or medical), Charlson's comorbidity index,^(^^16^^)^ infection source, number and type of organ dysfunction (respiratory, renal, platelet, hepatic, hemodynamic and neurological), and the need for hemodialysis.

Organ dysfunction was characterized as defined by the SSC:^(^^9^^)^ respiratory dysfunction: arterial hypoxemia (partial pressure arterial oxygen/fraction of inspired oxygen - PaO_2_/FiO_2_ < 300); renal dysfunction: acute oliguria (urine output < 0.5mL/kg/h for at least 2 hours) or an increase in creatinine levels > 0.5mg/dL; platelet dysfunction: platelet count < 100.000µ/L; hepatic dysfunction: total bilirubin > 4mg/dL; hemodynamic dysfunction: MAP < 70mmHg or systolic blood pressure < 90mmHg or systolic blood pressure decrease > 40mmHg; and neurological dysfunction: any alteration in the level of consciousness.

The primary outcomes were in-hospital mortality. The mortality at 30 days, length of ICU stay and length of hospital stay were considered secondary outcomes.

### Statistical analysis

Data on risk factors associated with the main outcome were tabulated and analyzed using descriptive statistics methods including proportions, means and standard deviations. The association of the continuous variables with the main outcome and exposure factor was performed by Student's t-test and categorical variables by the chi-square test. Mantel-Haenszel estimates were used to define possible confounding variables and odds ratio homogeneity tests were used to define possible effect-modifying variables considering a significance level of 5%. Variables that altered the crude effect of the exposure variable on the main outcome by more than 20% were considered as possible effect confounders and were included in the logistic regression, as well as those that presented nonhomogeneous odds ratios. The likelihood ratio test was used for inclusion and maintenance of the variable in the logistic regression model considering a significance level of 5%.

The study protocol was approved by the Ethics Committee of *Hospital Municipal São José de Joinville* under registration number CAAE 51661515.3.0000.5362.

## RESULTS

A total of 543 hospitalized patients diagnosed with sepsis between 2010 and 2015 were included. The mean frequency was 90.5 (85 to 105) cases/year. Of these, 319 (58.8%) patients had hospital-acquired sepsis.

Table 2 shows the characteristics of the groups. There was no difference in time between changes in vital signs and diagnosis (0:55 minutes *versus* 1:25 hours between community and hospital sepsis, respectively, p = 0.06). The most prevalent sources of infection were the lungs, urinary tract and abdomen. Patients with hospital-acquired sepsis had a higher frequency of abdominal (p = 0.02) and bloodstream infections (p < 0.001). This group also exhibited more severe disease according to the APACHE II score (p < 0.001), had a greater need for hemodialysis (p = 0.02), and had a larger number of organ dysfunctions, particularly respiratory (p < 0.001) and neurological dysfunctions (p < 0.001).

**Table 2:** Characteristics of hospitalized patients with sepsis of community and hospital origin

Characteristics	Community sepsis (n = 224)	Hospital sepsis (n = 319)	p value
Male	107 (47.7)	151 (47.3)	0.92
Age (years)	60 (58 - 63)	62 (60 - 66)	0.79
APACHE II score (points)	17 (15 - 19)	20 (19 - 21)	< 0.001
Charlson's index (points)*	2 (2 - 3)	3 (2 - 3)	0.46
Surgical hospitalization	24 (10.7)	105 (32.9)	< 0.001
Time between change in vital signs and diagnosis (hours)	0:55 (0:27 - 1:22)	1:25 (0:52 - 1:30)	0.06
Infection source			
Pulmonary	65 (29.0)	92 (28.8)	0.96
Urinary	73 (32.5)	74 (23.1)	0.01
Abdominal	33 (14.7)	72 (22.5)	0.02
Central nervous system	2 (0.9)	3 (0.9)	0.95
Soft tissue	17 (7.5)	20 (6.2)	0.54
Bloodstream	3 (1.3)	21 (6.5)	< 0.001
Other	30 (13.3)	35 (10.9)	0.39
Hemodialysis	27 (12.0)	62 (19.4)	0.02
Organ dysfunction			
Respiratory dysfunction	75 (33.4)	163 (51.0)	< 0.001
Renal insufficiency	82 (36.6)	128 (40.1)	0.40
Platelet dysfunction	71 (31.6)	81 (25.3)	0.11
Hepatic dysfunction	34 (15.1)	56 (17.5)	0.46
Hemodynamic instability	117 (52.2)	189 (59.2)	0.10
Neurological dysfunction	71 (31.6)	154 (48.2)	< 0.001

* Charlson's index, weighted index that evaluates the 10-year mortality risk in patients with several comorbidities. APACHE II - Acute Physiology and Chronic Health Disease Classification System II. Results expressed as n (%), mean (standard deviation), or interquartile range.

Adherence to the different resuscitation guidelines is shown in table 3. As seen in the table, adherence to the complete resuscitation bundle was present in 34.8% of patients with community-acquired sepsis and in 41.4% with hospital-acquired sepsis (p = 0.12). No differences were observed for the other components of the resuscitation bundle.

**Table 3:** Frequency of application of resuscitation bundle items in patients with sepsis of community and hospital origin

Bundle items	Community (n = 224)	Hospital (n = 319)	p value
Antibiotic in less than 1 hour	140 (62.5)	186 (58.3)	0.33
Obtaining blood cultures before antibiotic	184 (82.1)	245 (76.8)	0.13
Obtaining initial lactate	210 (93.7)	285 (89.3)	0.07
Fluid resuscitation/vasopressors if MAP < 65mmHg	109/117 (93.1)	173/196 (88.2)	0.16
Complete resuscitation bundle	78 (34.8)	132 (41.3)	0.12

MAP - mean arterial pressure. The results are expressed as n (%).

The outcomes of the patients are shown in table 4. The overall in-hospital mortality was 24.4% (n = 133) and was higher in patients with hospital-acquired sepsis compared to those with community-acquired sepsis (30.7% *versus* 15.6%; p < 0.001). The group with hospital-acquired sepsis had longer hospital [8 (8 - 10) *versus* 23 (20 - 27) days; p < 0.001] and ICU lengths of stay [5 (4 - 7) *versus* 8.5 (7 - 10); p < 0.001]. The time of hospitalization after diagnosis of sepsis was also higher in patients with hospital-acquired sepsis [8 (8 - 10) *versus* 13 (7 - 24) days; p < 0.001].

**Table 4:** Outcomes of patients diagnosed with community- and hospital-acquired sepsis

Outcomes	Community (n = 224)	Hospital (n = 319)	p value
Length of stay (days)			
In the ICU	5 (4 - 7)	8.5 (7 - 10)	< 0.001
After diagnosis of sepsis	8 (8 - 10)	13 (7 - 24)	< 0.001
At the hospital	8 (8 - 10)	23 (20 - 27)	< 0.001
Mortality			
30 days	29 (12.9)	60 (18.8)	0.07
Hospital	35 (15.6)	98 (30.7)	< 0.001

ICU - intensive care unit. The results are expressed as the median (interquartile range) or n (%).

Logistic regression was performed to evaluate the association between clinically significant variables (community/hospital sepsis, hemodialysis, respiratory dysfunction, hemodynamic instability, length of stay in the ICU, age, APACHE II score, and neurological dysfunction) and the risk of death (Table 1S - Supplementary material). After adjusting for age, APACHE II score, and hemodynamic and respiratory dysfunction, hospital-acquired sepsis remained associated with increased mortality (OR 1.96; 95%CI 1.15 - 3.32; p = 0.013).

## DISCUSSION

Our results show that hospital-acquired sepsis is associated with higher mortality and longer ICU and hospital stays compared to community-acquired sepsis. These results corroborate the few studies comparing community-acquired and hospital-acquired sepsis, showing that the latter is associated with poorer outcomes.^(^^14,17-19^^)^

We observed a frequency of community-acquired sepsis of 42%, in contrast to the findings of two other studies that reported a predominance of community-acquired sepsis cases (57.0% and 55.8%).^(^^17,20^^)^ These differences can be explained by the epidemiological particularities of each institution, such as patient age, severity, and presence of comorbidities. Regardless of the agreement of the findings between studies, epidemiological knowledge is of clinical importance and has implications for the development of early identification strategies for patients with sepsis. On the other hand, there is agreement between our findings and the other studies regarding mortality and length of hospital and ICU stay, which were higher among patients with hospital-acquired sepsis.^(^^14,17-20^^)^ In addition, the sites of infection were similar among the different studies, with a prevalence of pulmonary and abdominal infections.^(^^17,20^^)^ Corroborating the findings of Page et al.,^(^^14^^)^ we observed a clear predominance of abdominal infections and infections resulting from surgical procedures among the cases of hospital-acquired sepsis, a fact that can help define specific strategies for the detection of a characteristic phenotype of nosocomial sepsis.^(^^14^^)^ In this respect, the present results suggest that surgical patients with a clinical suspicion of abdominal infection should be monitored closely since they are more likely to develop sepsis, at least at our institution.

Mortality was significantly higher among patients with hospital-acquired sepsis than among those with community-acquired sepsis (30.7% *versus* 15.6%; p < 0.001). Page et al.^(^^14^^)^ suggested that, in contrast to emergency departments, wards often do not have the necessary resources for the frequent monitoring of vital data and laboratory test results. If this is the case, specific strategies need to be implemented so that sepsis can also be detected early on the wards. In our hospital, we have an electronic warning system coupled to the electronic chart that allows the rapid detection of patients with sepsis both in the emergency room and in the ward (1:22 ± 3:28 hours *versus* 1:58 ± 3:41 hours; p = 0.06). Despite the strong tendency towards a mathematical difference in the time of detection between the two groups, the average difference of 36 minutes does not seem to be clinically relevant. Although sepsis is detected within less than 2 hours both on the wards and in the emergency room, mortality from hospital-acquired sepsis was higher than that associated with community-acquired sepsis, supporting the view that hospital-acquired sepsis occurs in intrinsically more severe patients. In addition, even after adjusting for confounding factors, such as age and severity, the risk of death continued to be significantly higher in the group with hospital-acquired sepsis, suggesting that intrinsic variables of the patients seem to have an effect on the risk of death associated with sepsis.

In the last 15 years, several studies conducted in emergency departments have shown that the early management of sepsis using resuscitation bundles is associated with a reduction in mortality.^(^^7,9-13^^)^ One may infer that aggressive strategies for compliance with the resuscitation bundle also contribute to reducing mortality in patients with hospital-acquired sepsis. Our results contradict this inference considering that there were no significant differences in adherence to the complete resuscitation bundle or in the time to diagnosis, and adherence to early antibiotic therapy and compliance with hemodynamic resuscitation were similar in the two groups (Table 3). In this respect, some authors suggest that there is not sufficient evidence to indicate that the components of the resuscitation bundle can alter the outcome.^(^^21-24^^)^ The Edusepsis study demonstrated a reduction of mortality in septic patients, although adherence to the resuscitation bundle had only increased from 5.3% to 10%.^(^^22^^)^ In 2011, our group published a before-and-after study involving emergency and ward patients who demonstrated an association between the speed of diagnosis of sepsis (34 ± 48 hours *versus* 11 ± 17 hours; p < 0.001) and mortality reduction (61.7% *versus* 38.2%; p < 0.001), while adherence to the 6-hour bundle remained constant (32.3% *versus* 28.7%; p = 0.55).^(^^23^^)^ Similar results have been reported by Shiramizo et al.^(^^24^^)^ In this context, some studies suggest that probably only the early use of antimicrobials is important when the general care of patients is suitable. A prospective, observational multicenter cohort study in 44 German ICUs showed that the delay in source control beyond 6 hours may have a major impact on patient mortality, and there was only indirect evidence regarding the impact of timing of antimicrobial therapy on sepsis mortality, despite poor compliance with sepsis guideline recommendations.^(^^25^^)^ A retrospective study involving 185 hospitals in the New York State Department of Health database showed that longer times to complete the three-hour treatment package for patients with sepsis and to administer broad-spectrum antibiotics were associated with higher risk-adjusted hospital mortality.^(^^26^^)^

Patients in the hospital-acquired sepsis group received more resuscitation fluids, but it was not possible to evaluate their relationship with the outcome because there were other confounding factors interfering with the results. Some authors suggest that early liberal fluids are harmful to sepsis patients because there is no optimal MAP target, which causes a wide variability in fluid input among large sepsis studies. In addition, there might be a high prevalence of non-fluid-responsive patients for whom the excess fluid may be harmful.^(^^27^^)^ However, the adverse effect of positive balance is more commonly seen after the initial rescue phase of resuscitation.^(^^28^^)^ In this study, we did not evaluate fluid infusion after the resuscitation phase or parameters of fluid responsiveness of the patients.

Overall mortality (24.4%) and mortality among patients with community-acquired (15.6%) and nosocomial sepsis (30.7%) were lower than the Brazilian mortality reported by the SPREAD study (55.7%) and PROGRESS study (57.4%).^(^^5,6^^)^ This difference might be explained in part by the private nature of the hospital where the study was conducted since better infrastructure conditions and human resources could have influenced the results. In the SPREAD study, a high mortality was observed in centers with less availability of resources, without the necessary infrastructure for the treatment of sepsis and a lack of ICU beds, resulting in inadequate treatment and a delay in the first dose of antibiotics.^(^^5^^)^ On the other hand, the early rates (prior to early detection strategies) of sepsis-related mortality in our hospital reported in previous studies^(^^23,29^^)^ were similar to the national rates. These findings suggest that early detection may influence the reduction in mortality related to both community-acquired and hospital-acquired sepsis.

Our study had some limitations. It was an observational study, and potentially unrecognized confounding variables may have influenced outcomes. Since it was conducted at a private hospital in southern Brazil, the results may not be generalized to the public health system or to other centers in the region and in Brazil. The selection, diagnosis and treatment of patients were performed following the SSC guidelines, and the new definitions published in 2016 were not taken into consideration.^(^^1,9,30,31^^)^ The evaluation of organ dysfunction was also performed using the SSC definitions instead of the Sepsis-related Organ Failure Assessment (SOFA). Another limitation was that the study did not discriminate patients with healthcare-associated sepsis, whose prevalence and severity differed from community-acquired and hospital-acquired sepsis.^(^^14^^)^ The inclusion criteria did not consider patients who were readmitted during the period. Since the data were collected based on the electronic vital sign chart, some clinical signs of infection and organ dysfunction were not used for screening the patients, and it was not possible to classify patients with septic shock based on serum lactate levels. Another limitation was that we did not evaluate the cause of hospitalization for patients with hospital-acquired sepsis. Despite these limitations, the scarcity of literature and data on this topic highlights the importance of this study, which describes the profile of these two groups of patients and their hospital outcomes, reinforcing the need to adopt and maintain strategies for early detection.

## CONCLUSION

Hospital-acquired sepsis is associated with poorer outcomes, including higher mortality and longer intensive care unit and hospital stays, compared to community-acquired sepsis. Mortality was higher in patients with hospital-acquired sepsis despite the same time to antibiotic administration and even more aggressive fluid resuscitation. Overall mortality in our sample was lower than the previously reported sepsis mortality rate in Brazil. Despite some limitations, the knowledge of the profile of these two groups of patients and their respective outcomes helps define strategies for the detection and treatment of patients with community-acquired and hospital-acquired sepsis.
