# Supplementary material for: Characteristics and outcomes of patients with community-acquired and hospital-acquired sepsis
Source: Rev Bras Ter Intensiva. 2019 Jan-Mar;31(1):71–8. doi: 10.5935/0103-507X.20190013 (PMC6443308; doi:10.5935/0103-507X.20190013)
Supplement: Supplementary file 1 [file rbti-31-01-0071-suppl1.pdf]

Characteristics and outcomes of patients with community-acquired and hospital-acquired sepsis

Características e desfechos de pacientes com sepse adquirida na comunidade e no hospital

Glauco Adrieno Westphal[1], Aline Braz Pereira[1], Silvia Maria Fachin[1], Ana Carolina Caldara Barreto[2], Ana Carolina Gern Junqueira Bornschein[2], Milton Caldeira Filho[1], Álvaro Koenig[1]

Table 1S - Factors associated with mortality due to sepsis during hospitalization

Variable	Total	Survivors n (%)	Nonsurvivors n (%)	OR (95% CI)	p value
Sepsis					
Hospital	319	221 (69.3)	98 (30.7)	0.40 (0.26 - 0.63)	< 0.001
Community	224	189 (84.4)	35 (15.6)		
Sex					
Male	258	193 (74.9)	65 (25.1)	1.07 (0.72 - 1.61)	0.72
Female	285	217 (76.2)	68 (23.8)		
Hospitalization					
Surgical	129	95 (73.7)	34 (26.3)	0.85 (0.54 - 1.35)	0.49
Clinical	414	318 (76.9)	96 (23.1)		
Age, years					
≥ 65	264	171 (64.8)	93 (35.2)	3.33 (2.17 - 5.26)	< 0.001
< 65	279	239 (85.7)	40 (14.3)		
APACHE II score					
≥ 20	241	154 (64)	87 (36.0)	4.16 (2.63 - 6.66)	< 0.001
< 20	302	261 (86.5)	41 (13.5)		
Length of stay in the ICU (days)					
≥ 14	81	44 (54.4)	37 (45.6)	1.96 (1.14 - 3.33)	0.01
< 14	224	156 (69.6)	68 (30.4)		
Respiratory dysfunction					
Yes	238	136 (57.2)	102 (42.8)	7.69 (4.54 - 12.5)	< 0.001
No	305	277 (90.9)	28 (9.1)		
Hemodialysis					
Yes		52 (58.5)	37 (41.5)	2.77 (1.66 - 4.54)	< 0.001
No	454	362 (79.8)	92 (20.2)		
Platelet dysfunction					
Yes	152	105 (69.1)	47 (30.9)	1.58 (1.04 - 2.43)	0.03
No	391	308 (78.8)	83 (21.2)		
Hemodynamic instability					
Yes	306	205 (67.0)	101 (33.0)	3.33 (2.04 - 5.26)	< 0.001
No	237	208 (87.8)	29 (12.2)		
Hepatic dysfunction					
Yes	90	57 (63.4)	33 (36.6)	2.04 (1.25 - 3.33)	0.004
No	453	356 (78.6)	97 (21.4)		

Continue...

... continuation

Neurological dysfunction					
Yes	225	138 (61.4)	87 (38.6)	3.84 (2.5 - 6.25)	< 0.001
No	318	275 (86.5)	43 (13.5)		
Renal insufficiency					
Yes	210	135 (64.3)	75 (35.7)	2.77 (1.78 - 4.16)	< 0.001
No	333	278 (83.5)	55 (16.5)		
Fluid resuscitation					
Yes	438	321 (73.3)	117 (26.7)	2.0 (1.11 - 3.57)	0.02
No	105	89 (84.8)	16 (15.2)		
Start of antibiotic therapy (hour)					
> 1	217	180 (83.0)	37 (17.0)	0.93 (0.59 - 1.44)	0.73
≤ 1	326	248 (76.1)	78 (23.9)		

APACHE II score - Acute Physiology and Chronic Health Disease Classification System II; ICU - intensive care unit.
